# Interactions of *Cryptococcus* with Dendritic Cells

**DOI:** 10.3390/jof4010036

**Published:** 2018-03-15

**Authors:** Karen L. Wozniak

**Affiliations:** Department of Microbiology and Molecular Genetics, Oklahoma State University, Stillwater, OK 74078, USA; Karen.Wozniak@okstate.edu; Tel.: +1-405-744-7914

**Keywords:** dendritic cell, *Cryptococcus neoformans*, *Cryptococcus gattii*, innate immunity, fungal immunity

## Abstract

The fungal pathogens *Cryptococcus neoformans* and *Cryptococcus gattii* can cause life-threatening infections in immune compromised and immune competent hosts. These pathogens enter the host via inhalation, and respiratory tract innate immune cells such as dendritic cells (DCs) are one of the first host cells they encounter. The interactions between *Cryptococcus* and innate immune cells play a critical role in the progression of disease in the host. This review will focus specifically on the interactions between *Cryptococcus* and dendritic cells (DCs), including recognition/processing by DCs, effects of immune mediators on DC recruitment and activity, and the potential for DC vaccination against cryptococcosis.

## 1. Dendritic Cells

Dendritic cells (DCs) are innate phagocytes in the lung, and they function as sentinels of the immune system. Due to their presence in the lung, they are one of the first cell types to encounter inhaled pathogens such as *Cryptococcus*. These cells can not only recognize, phagocytose, and degrade invading pathogens, but also present antigen to naïve T cells and ultimately direct the adaptive immune response. DCs can be affected by the local cytokine mileu, and their recruitment to the site of infection often correlates with pathogen control. Early studies examining the DC response to *Cryptococcus* showed that recruitment of specific subsets of DCs, including Langerhans and myeloid DCs, are associated with protective responses [1].

## 2. Recognition of *Cryptococcus* by DCs

In order to initiate immune responses, DCs must first recognize cryptococcal cells in the lung. Cryptococcal cells are surrounded by an anti-phagocytic capsule mainly comprised of glucuronoxylomannan (GXM) and galactoxylomannan (GalXM), and the capsule allows the fungi to evade detection by phagocytes [2,3,4]. However, once opsonized by complement or by anti-capsular antibody, the cells can be phagocytosed by DCs [5]. Studies examining toll-like receptors (TLR2 and TLR4) on DCs show that while these receptors can recognize cryptococcal capsular component GXM [6,7], they play a minor role in protection against *C. neoformans* infection [8]. TLR2^−/−^ and TLR4^−/−^ mice produce similar levels of interleukin 1β (IL-1β), IL-6, IL-12p40 and tumor necrosis factor-α (TNF-α) compared to littermates during cryptococcal infection, and survival rates are similar compared to wild type (WT) mice [6,8]. DNA from *Cryptococcus neoformans* can be recognized by TLR9 in myeloid DCs and can activate these DCs [9]. Later studies identified a specific *URA5* gene fragment of cryptococcal DNA that can activate bone marrow-derived DCs (BMDCs) in a TLR9-dependent manner [10]. Additionally, although DNA of *C. neoformans* activates BMDCs via TLR9 [9], the culture supernatants from *Cryptococcus neoformans* suppress activation of BMDCs stimulated with cryptococcal DNA, but not with DNA from other fungi [11]. Mice lacking TLR9 have an increased susceptibility to *C. neoformans* infection, and TLR9 appears to be required for recruitment of DCs to the site of infection [9,12,13] (Table 1). In addition to TLRs, c-type lectin receptors have been implicated in DC recognition of several fungal pathogens (reviewed in [14]). However, in contrast to other fungal pathogens, recent studies have shown that conventional DCs do not recognize *C. neoformans* by c-type lectin receptors such as Dectin-1, Dectin-2, or Dectin-3 [15,16,17]. The mannose receptor (MR) on DCs can recognize cryptococcal mannoprotein (MP), and MP activates and induces maturation of human DCs by a process that involves recognition by the MR [18,19] (Table 1). Furthermore, after pulmonary cryptococcal infection, MR^−/−^ mice have increased fungal burden compared to WT mice, and DCs from MR^−/−^ mice do not induce *Cryptococcus*-specific T cell proliferation when compared to DCs from WT mice [20].

## 3. Cryptococcal Capsular and Cell Wall Components Affect Dendritic Cell Maturation and Activation

The surface molecules of *Cryptococcus*, including capsular materials, mannoproteins, and chitin, can affect dendritic cell maturation and activation. Although the cryptococcal capsule is anti-phagocytic, it can also have direct effects on dendritic cells. In studies examining interactions of human DCs incubated in vitro with encapsulated vs. acapsular strains, DCs incubated with the encapsulated strain did not up-regulate surface expression of major histocompatibility complex (MHC) class I or class II or CD83, although incubation with both strains induced surface expression of CD40 [33]. However, in another study using a murine bone marrow-derived DC cell line (D1) with a WT cryptococcal strain compared to an acapsular mutant, incubation with the acapsular strain induced expression of genes involved in DC maturation, including MHC II, complement component C3, IL-12, IL-2, IL-1α, IL-1β, IL-6, IL-10, TNF-α, CC-chemokine receptor 7 (CCR7), CC-chemokine ligand 17 (CCL17), CCL22, CCL3, CCL4, CCL7, and C-X-C motif chemokine 10 (CXCL10). In contrast, the encapsulated *C. neoformans* strain did not induce these genes to be expressed in DCs [34]. Cryptococcal mannoproteins can also affect DC maturation and activation, even though these are sometimes masked by the presence of capsule. Studies have also shown that cryptococcal mannoproteins can induce the surface expression of MHC class I and II on human DCs, as well as costimulatory molecules including CD40, CD86, and CD83 [18]. Another part of the fungal cell wall, chitin, is not directly recognized by DCs, but it can affect DC-initiated T cell polarization [35]. Indirect chitin recognition via chitotriosidase leads to differentiation of non-protective Th2-type T cells by lung-resident interferon regulatory factor 4 (IRF4)-dependent conventional DCs.

## 4. Cryptococcal Antifungal Activity by DCs

Following recognition, for efficient uptake by DCs, encapsulated *Cryptococcus* must be opsonized with antibody or complement before phagocytosis can occur [5,33]. Following uptake and processing, DCs present antigen to *Cryptococcus*-specific T cells, and can thus initiate the adaptive immune response [36]. Once phagocytosed by DCs, cryptococcal cells can quickly move into the endolysosomal pathway, and these compartments acquire lysosomal markers such as lysosomal-associated membrane protein 1 (LAMP-1) [37] and also recruit the tetraspanin CD63 to the acidified compartments [38]. Additional studies showed that lysosomal extract from murine BMDCs and human peripheral blood mononuclear cell (PBMC)-derived DCs has anti-cryptococcal activity [37], and specific DC lysosomal enzymes found in the DC lysosomal extract, such as Cathepsin B, have anticryptococcal activity [39]. Cathepsin B treatment of *Cryptococcus* leads to a hole in the fungal cell wall, and osmotic lysis kills the organism [39]. Further studies examining fractions of DC lysosomal extract showed that several different molecular weight fractions have anti-cryptococcal activity [40], and lysosomal components with antifungal activity are being identified. Following uptake and degradation of *C. neoformans*, the DCs upregulate surface molecules involved in costimulation and antigen presentation (MHC II, CD80, CD86) and are capable of antigen presentation to T cells [36]. Although uptake, processing, and killing by DCs occurs with all cryptococcal species, *C. gattii* can suppress further DC-mediated T cell activation. Human DCs can phagocytose and even kill *C. gattii* strain R265 [41], but due to the *C. gattii* capsule, the DCs fail to upregulate surface markers associated with maturation, such as CD86, CD83, MR, and CD32, and fail to present cryptococcal antigen to T cells compared to DCs that encounter the acapsular *C. gattii* mutant, cap 59 [42]. DC maturation requires extracellular receptor signaling that is dependent on TNF-α and p38 MAPK, which occurs following interaction of the acapsular mutant with human DCs but does not occur following interaction of the encapsulated *C. gattii* strain with human DCs [42]. Additional studies showed that *C. gattii* strain JP02 interaction with the JAWS II DC cell line (a murine bone marrow-derived DC cell line) results in decreased production of cytokines such as IL-6, IL-12, and TNF-α compared to the JAWS II DCs exposed to *C. neoformans* H99 [43]. This was due to the alteration in one of two *O*-acetyl groups in the GXM of *C. gattii* JP02 compared to *C. neoformans* H99. Therefore, this change in the GXM structure resulted in impaired pathogen recognition of *C. gattii* compared to *C. neoformans* by the JAWS II DC cell line [43]. The interaction of plasmacytoid DCs (pDCs) with *C. neoformans* involves different antifungal mechanisms than that of conventional DCs. Recognition and uptake of *C. neoformans* by pDCs requires Dectin-3, and anti-cryptococcal activity is due to the production of reactive oxygen species (ROS) by pDCs [44]. Murine pDCs have direct anti-cryptococcal activity via ROS, and this activity is independent of nitric oxide (pDCs from iNOS^−/−^ mice still have activity). In addition, recruitment of murine pDCs to the lungs during cryptococcal infection requires C-X-C motif chemokine receptor 3 (CXCR3) [44]. Human pDCs also inhibit cryptococcal growth, but this activity is not as dependent on ROS, and while Dectin-3 is necessary for optimal inhibition of cryptococcal growth, it is not required for cryptococcal uptake by human pDCs [44].

## 5. Influence of Immune Mediators on DC Recruitment and Activity

Production of immune mediators by the host during cryptococcal infection, including cytokines, chemokines and scavenger receptors, can affect DC recruitment and/or DC activation (Table 1). Blocking of these mediators by using knockout (KO) mice or neutralization can lead to differences in DC recruitment to the lung, DC activation/maturation, and DC anti-cryptococcal activity. Studies examining the Th1-type cytokines have shown that the Th1-type immune response is important for cryptococcal clearance as well as DC recruitment and polarization (reviewed in [45,46,47]). Following protective immunization with an IFN-γ-producing cryptococcal strain (H99γ), mice displayed a predominately Th1-type protective response [21,48]. During challenge, protectively immunized mice had an early infiltration of DCs to the lungs at day 3 post-challenge [21]. Studies examining Th17-type cytokines have shown differential responses depending on the model and cryptococcal strain used. IL-17A is elevated in lung homogenates of protectively immunized mice following immunization with the interferon-gamma producing strain of H99 (H99γ) during primary infection and during challenge with wild-type H99. Despite elevated IL-17A in the lungs of these mice, the presence of the cytokine and signaling through the IL-17 receptor are not necessary for protection [22]. In addition, the absence of IL-17A did not alter DC recruitment to the lung [22]. However, other studies have shown that IL-17A can enhance anti-cryptococcal responses to *C. neoformans* strain 52D by increased infiltration of conventional DCs to the pulmonary tissues as well as increased co-stimulatory molecule expression on DCs [23]. In addition, the maturation of DCs is altered in IL-17A^−/−^ mice [23]. The IL-17^−/−^ mice have reduced expression of MHC II, CD80 and CD86 on DCs at 4–8 weeks post-cryptococcal infection compared to WT mice [23]. The observed differences in the role of IL-17A may be due to the differences in mouse models (BALB/c vs. C57Bl/6) and the cryptococcal strain (H99 vs. 52D) used in these studies. Although the Th2-type cytokine response has been associated with exacerbation of cryptococcal disease, more recent studies have shown that Th2-type cytokines may be important in early stages of cryptococcal control and may be involved in DC recruitment and activation. In IL-4 receptor alpha^−/−^ mice infected with *C. neoformans* serotype D, the mice have reduced pulmonary recruitment of DCs and reduced DC gene expression of CCL2 and CCL20 [24]. In addition, BMDCs stimulated in vitro with *C. neoformans* and IL-4 have increased production of IL-12p70 and decreased production of IL-10 in the culture supernatants compared to BMDCs stimulated with *C. neoformans* alone [24]. Production of the regulatory cytokine IL-10 can be associated with persistent cryptococcal infections, and blocking IL-10 late during cryptococcal infection leads to increased accumulation and activation of pulmonary DCs [25]. The chemokine GM-CSF is important in differentiation and activation of pulmonary DCs, and GM-CSF^−/−^ mice have deficiencies in lung leukocyte recruitment, Th2 and Th17 responses, and have defective recruitment of DCs and macrophages to the lung during pulmonary cryptococcal infection [26]. During cryptococcal infection, GM-CSF^−/−^ mice have increased numbers of Ly6C^hi^ macrophage and DC precursors in the blood and in the lungs, but these cells do not differentiate or migrate to the lung during cryptococcal infection [26]. In contrast to what is observed in *C. neoformans* infections, *C. gattii* infection dampens both Th1 and Th17 responses, and it appears to do this in part by attenuating DC function [27]. When BMDCS are cultured with *C. gattii* or *C. neoformans* and then cultured with naïve CD4^+^ T cells, the T cells cultured with the *C. gattii-*exposed DCs produce less IFN-γ and IL-17A than those cultured with DCs exposed to *C. neoformans*. In addition, BMDCs cultured with *C. gattii* have reduced surface expression of MHC II, and gene expression for IL-12 and IL-23 is significantly reduced compared to BMDCs cultured with *C. neoformans* [27].

In addition to cytokines and chemokines, cell surface receptors on DCs, including chemokine receptors and scavenger receptors, have been implicated in DC maturation and recruitment during cryptococcal infection. The chemokine receptor CCR2 has been shown to mediate the accumulation of DCs in the lungs of *C. neoformans* infected mice [28]. In CCR2^−/−^ mice infected with *C. neoformans* strain 52D, mice have reduced fungal clearance, which is associated with reduced accumulation of lung DCs [29]. In addition, these studies showed that the Ly-6C^hi^ monocyte population differentiates into lung conventional DCs during cryptococcal infection, and in CCR2^−/−^ mice, the Ly-6C^hi^ DC precursor population accumulates in the bone marrow, suggesting that the reduced lung DC numbers are due to the lack of trafficking of DC precursor cells to the lung during cryptococcal infection [29]. Scavenger receptor A (SRA)^−/−^ mice have an increased accumulation of DCs in the lungs during cryptococcal infection, and these DCs have enhanced surface expression of co-stimulatory molecules CD80 and CD86, which leads to improved fungal clearance [30]. Expression of the scavenger receptor MARCO causes early accumulation and alternative activation of conventional DCs in lung associated lymph nodes, and its expression alters the Th1/Th2 cytokine balance to promote persistent infection and dissemination [31]. Impaired accumulation of lung DCs occurs during cryptococcal infection in MARCO^−/−^ mice, and these DCs have decreased expression of CD206, but increased expression of the costimulatory molecules CD80 and CD86 [31]. MARCO deficiency leads to impaired fungal control and impaired recruitment of pulmonary conventional DCs [32]. Furthermore, MARCO^−/−^ DCs have reduced phagocytosis of *C. neoformans* and indirectly reduced fungal killing, which is associated with the modulation of the cytokine environment in these mice [32].

## 6. Dendritic Cell Vaccines against Cryptococcosis

Thus far, the most promising dendritic cell-based vaccine for cryptococcosis involves targeting glucan particles to DCs [49,50]. These glucan particles contain alkaline extracts from cryptococcal strains lacking capsule or chitosan and can be recognized by Dectin-1 on DCs [51]. Immunization of mice with these glucan particles leads to production of antigen-specific antibody and generation of protective Th1-type and Th17-type T cell responses [52,53]. Mice receiving these glucan particle-based *C. neoformans* vaccinations have increased survival rates after *C. neoformans* or *C. gattii* challenge compared control animals [49,50]. Another *C. gattii* vaccine strategy has used BMDCs pulsed with an acapsular *C. gattii* strain, Δ cap 60 [54]. Mice that were immunized via tail vein with the pulsed BMDCs were protected from pulmonary challenge with *C. gattii* R265. These DC-immunized mice had less severe pathology, lower fungal burden, and increased survival compared to non-immunized mice [54]. The protectively immunized mice also had increases in IL-17A, IFN-γ, and TNF-α producing lymphocytes in the spleen and lung. The DC *C. gattii* vaccine also induces multinucleated giant cells, and its efficacy is partially reduced in IFN-γ^−/−^ mice, suggesting that the protection generated depends on IFN-γ production [55]. These studies suggest that DC vaccination strategies for *C. neoformans* and *C. gattii* infections are promising strategies for protection against cryptococcosis.

## 7. Conclusions

DCs are important first-responders during cryptcococcal infection in the lung. They have roles in recognition, uptake, and degradation of *Cryptococcus*. In addition, DCs and their maturation and downstream T cell activating capacity are influenced by fungal factors, such as capsular materials and fungal cell wall components, and they can also be influenced by immune mediators. All of these factors can result in different responses for the host, either clearance of cryptococcal infection or persistent infection. Understanding the DC interactions with *Cryptococcus* may lead to immunotherapies and effective DC-based cryptococcal vaccines in the future.

## Figures and Tables

**Table 1:** Pattern recognition receptors (PRR)/Immune mediator effects on dendritic cell (DC) recruitment and activation.

PRR/Immune Mediator	DC Recruitment	DC Activation	Reference
TLR9	+	+	[9,10,11,12,13]
MR		+	[18,19]
Th1-type cytokines	+	+	[21]
IL-17A	−/+	−/+	[22,23]
IL-4	+		[24]
IL-10	−	−	[25]
GM-CSF	+	+	[26,27]
CCR2	+		[28,29]
SRA	−	−	[30]
MARCO	+	−	[31,32]

TLR: toll-like receptor; MR: mannose receptor; Th: T helper cell; IL: interleukin; GM-CSF: granulocyte-macrophage colony-stimulating factor; CCR: C-C chemokine receptor; SRA: scavenger receptor A; MARCO: macrophage receptor with collagenous structure.

## References

[1] Bauman S.K., Nichols K.L., Murphy J.W. (2000). Dendritic cells in the induction of protective and nonprotective anticryptococcal cell-mediated immune responses. J. Immunol..

[2] Kozel T.R., Gotschlich E.C. (1982). The capsule of *Cryptococcus neoformans* passively inhibits phagocytosis of the yeast by macrophages. J. Immunol..

[3] Kozel T.R., Pfrommer G.S., Guerlain A.S., Highison B.A., Highison G.J. (1988). Role of the capsule in phagocytosis of *Cryptococcus neoformans*. Rev. Infect. Dis..

[4] Vecchiarelli A., Pericolini E., Gabrielli E., Kenno S., Perito S., Cenci E., Monari C. (2013). Elucidating the immunological function of the *Cryptococcus neoformans* capsule. Future Microbiol..

[5] Kelly R.M., Chen J., Yauch L.E., Levitz S.M. (2005). Opsonic requirements for dendritic cell-mediated responses to *Cryptococcus neoformans*. Infect. Immun..

[6] Yauch L.E., Mansour M.K., Shoham S., Rottman J.B., Levitz S.M. (2004). Involvement of CD14, toll-like receptors 2 and 4, and MyD88 in the host response to the fungal pathogen *Cryptococcus neoformans* in vivo. Infect. Immun..

[7] Shoham S., Huang C., Chen J.M., Golenbock D.T., Levitz S.M. (2001). Toll-like receptor 4 mediates intracellular signaling without TNF-α release in response to *Cryptococcus neoformans* polysaccharide capsule. J. Immunol..

[8] Nakamura K., Miyagi K., Koguchi Y., Kinjo Y., Uezu K., Kinjo T., Akamine M., Fujita J., Kawamura I., Mitsuyama M. (2006). Limited contribution of toll-like receptor 2 and 4 to the host response to a fungal infectious pathogen, *Cryptococcus neoformans*. FEMS Immunol. Med. Microbiol..

[9] Nakamura K., Miyazato A., Xiao G., Hatta M., Inden K., Aoyagi T., Shiratori K., Takeda K., Akira S., Saijo S. (2008). Deoxynucleic acids from *Cryptococcus neoformans* activate myeloid dendritic cells via a TLR9-dependent pathway. J. Immunol..

[10] Tanaka M., Ishii K., Nakamura Y., Miyazato A., Maki A., Abe Y., Miyasaka T., Yamamoto H., Akahori Y., Fue M. (2012). Toll-like receptor 9-dependent activation of bone marrow-derived dendritic cells by URA5 DNA from *Cryptococcus neoformans*. Infect. Immun..

[11] Yamamoto H., Abe Y., Miyazato A., Tanno D., Tanaka M., Miyasaka T., Ishii K., Kawakami K. (2011). *Cryptococcus neoformans* suppresses the activation of bone marrow-derived dendritic cells stimulated with its own DNA, but not with DNA from other fungi. FEMS Immunol. Med. Microbiol..

[12] Zhang Y., Wang F., Bhan U., Huffnagle G.B., Toews G.B., Standiford T.J., Olszewski M.A. (2010). TLR9 signaling is required for generation of the adaptive immune protection in *Cryptococcus neoformans*-infected lungs. Am. J. Pathol..

[13] Wang J.P., Lee C.K., Akalin A., Finberg R.W., Levitz S.M. (2011). Contributions of the MyD88-dependent receptors IL-18r, IL-1R, and TLR9 to host defenses following pulmonary challenge with *Cryptococcus neoformans*. PLoS ONE.

[14] Hardison S.E., Brown G.D. (2012). C-type lectin receptors orchestrate antifungal immunity. Nat. Immunol..

[15] Nakamura K., Kinjo T., Saijo S., Miyazato A., Adachi Y., Ohno N., Fujita J., Kaku M., Iwakura Y., Kawakami K. (2007). Dectin-1 is not required for the host defense to *Cryptococcus neoformans*. Microbiol. Immunol..

[16] Nakamura Y., Sato K., Yamamoto H., Matsumura K., Matsumoto I., Nomura T., Miyasaka T., Ishii K., Kanno E., Tachi M. (2015). Dectin-2 deficiency promotes Th2 response and mucin production in the lungs after pulmonary infection with *Cryptococcus neoformans*. Infect. Immun..

[17] Campuzano A., Castro-Lopez N., Wozniak K.L., Leopold Wager C.M., Wormley F.L. (2017). Dectin-3 is not required for protection against *Cryptococcus neoformans* infection. PLoS ONE.

[18] Pietrella D., Corbucci C., Perito S., Bistoni G., Vecchiarelli A. (2005). Mannoproteins from *Cryptococcus neoformans* promote dendritic cell maturation and activation. Infect. Immun..

[19] Mansour M.K., Latz E., Levitz S.M. (2006). *Cryptococcus neoformans* glycoantigens are captured by multiple lectin receptors and presented by dendritic cells. J. Immunol..

[20] Dan J.M., Wang J.P., Lee C.K., Levitz S.M. (2008). Cooperative stimulation of dendritic cells by *Cryptococcus neoformans* mannoproteins and CpG oligodeoxynucleotides. PLoS ONE.

[21] Wozniak K.L., Ravi S., Macias S., Young M.L., Olszewski M.A., Steele C., Wormley F.L. (2009). Insights into the mechanisms of protective immunity against *Cryptococcus neoformans* infection using a mouse model of pulmonary cryptococcosis. PLoS ONE.

[22] Wozniak K.L., Hardison S.E., Kolls J.K., Wormley F.L. (2011). Role of IL-17A on resolution of pulmonary *C. neoformans* infection. PLoS ONE.

[23] Murdock B.J., Huffnagle G.B., Olszewski M.A., Osterholzer J.J. (2014). Interleukin-17A enhances host defense against cryptococcal lung infection through effects mediated by leukocyte recruitment, activation, and gamma interferon production. Infect. Immun..

[24] Grahnert A., Richter T., Piehler D., Eschke M., Schulze B., Muller U., Protschka M., Kohler G., Sabat R., Brombacher F. (2014). IL-4 receptor-α-dependent control of *Cryptococcus neoformans* in the early phase of pulmonary infection. PLoS ONE.

[25] Murdock B.J., Teitz-Tennenbaum S., Chen G.H., Dils A.J., Malachowski A.N., Curtis J.L., Olszewski M.A., Osterholzer J.J. (2014). Early or late IL-10 blockade enhances th1 and Th17 effector responses and promotes fungal clearance in mice with cryptococcal lung infection. J. Immunol..

[26] Chen G.H., Teitz-Tennenbaum S., Neal L.M., Murdock B.J., Malachowski A.N., Dils A.J., Olszewski M.A., Osterholzer J.J. (2016). Local GM-CSF-dependent differentiation and activation of pulmonary dendritic cells and macrophages protect against progressive cryptococcal lung infection in mice. J. Immunol..

[27] Angkasekwinai P., Sringkarin N., Supasorn O., Fungkrajai M., Wang Y.-H., Chayakulkeeree M., Ngamskulrungroj P., Angkasekwinai N., Pattanapanyasat K. (2014). *Cryptococcus gattii* infection dampens Th1 and Th17 responses by attenuating dendritic cell function and pulmonary chemokine expression in the immunocompetent hosts. Infect. Immun..

[28] Osterholzer J.J., Curtis J.L., Polak T., Ames T., Chen G.-H., McDonald R., Huffnagle G.B., Toews G.B. (2008). CCR2 mediates conventional dendritic cell recruitment and the formation of bronchovascular mononuclear cell infiltrates in the lungs of mice infected with *Cryptococcus neoformans*. J. Immunol..

[29] Osterholzer J.J., Chen G.H., Olszewski M.A., Curtis J.L., Huffnagle G.B., Toews G.B. (2009). Accumulation of CD11b^+^ lung dendritic cells in response to fungal infection results from the CCR2-mediated recruitment and differentiation of Ly-6Chigh monocytes. J. Immunol..

[30] Qiu Y., Dayrit J.K., Davis M.J., Carolan J.F., Osterholzer J.J., Curtis J.L., Olszewski M.A. (2013). Scavenger receptor a modulates the immune response to pulmonary *Cryptococcus neoformans* infection. J. Immunol..

[31] Xu J., Flaczyk A., Neal L.M., Fa Z., Cheng D., Ivey M., Moore B.B., Curtis J.L., Osterholzer J.J., Olszewski M.A. (2017). Exploitation of scavenger receptor, macrophage receptor with collagenous structure, by *Cryptococcus neoformans* promotes alternative activation of pulmonary lymph node CD11b^+^ conventional dendritic cells and non-protective Th2 bias. Front. Immunol..

[32] Xu J., Flaczyk A., Neal L.M., Fa Z., Eastman A.J., Malachowski A.N., Cheng D., Moore B.B., Curtis J.L., Osterholzer J.J. (2017). Scavenger receptor marco orchestrates early defenses and contributes to fungal containment during cryptococcal infection. J. Immunol..

[33] Vecchiarelli A., Pietrella D., Lupo P., Bistoni F., McFadden D.C., Casadevall A. (2003). The polysaccharide capsule of *Cryptococcus neoformans* interferes with human dendritic cell maturation and activation. J. Leukoc. Biol..

[34] Lupo P., Chang Y.C., Kelsall B.L., Farber J.M., Pietrella D., Vecchiarelli A., Leon F., Kwon-Chung K.J. (2008). The presence of capsule in *Cryptococcus neoformans* influences the gene expression profile in dendritic cells during interaction with the fungus. Infect. Immun..

[35] Wiesner D.L., Specht C.A., Lee C.K., Smith K.D., Mukaremera L., Lee S.T., Lee C.G., Elias J.A., Nielsen J.N., Boulware D.R. (2015). Chitin recognition via chitotriosidase promotes pathologic type-2 helper T cell responses to cryptococcal infection. PLoS Pathog..

[36] Wozniak K.L., Vyas J.M., Levitz S.M. (2006). In vivo role of dendritic cells in a murine model of pulmonary cryptococcosis. Infect. Immun..

[37] Wozniak K.L., Levitz S.M. (2008). *Cryptococcus neoformans* enters the endolysosomal pathway of dendritic cells and is killed by lysosomal components. Infect. Immun..

[38] Artavanis-Tsakonas K., Love J.C., Ploegh H.L., Vyas J.M. (2006). Recruitment of CD63 to *Cryptococcus neoformans* phagosomes requires acidification. Proc. Natl. Acad. Sci. USA.

[39] Hole C.R., Bui H., Wormley F.L., Wozniak K.L. (2012). Mechanisms of dendritic cell lysosomal killing of *Cryptococcus*. Sci. Rep..

[40] Wozniak K.L. (2018). Oklahoma State University, Stillwater, OK, USA.

[41] Huston S.M., Li S.S., Stack D., Timm-McCann M., Jones G.J., Islam A., Berenger B.M., Xiang R.F., Colarusso P., Mody C.H. (2013). *Cryptococcus gattii* is killed by dendritic cells, but evades adaptive immunity by failing to induce dendritic cell maturation. J. Immunol..

[42] Huston S.M., Ngamskulrungroj P., Xiang R.F., Ogbomo H., Stack D., Li S.S., Timm-McCann M., Kyei S.K., Oykhman P., Kwon-Chung K.J. (2016). *Cryptococcus gattii* capsule blocks surface recognition required for dendritic cell maturation independent of internalization and antigen processing. J. Immunol..

[43] Urai M., Kaneko Y., Ueno K., Okubo Y., Aizawa T., Fukazawa H., Sugita T., Ohno H., Shibuya K., Kinjo Y. (2015). Evasion of innate immune responses by the highly virulent *Cryptococcus gattii* by altering capsule glucuronoxylomannan structure. Front. Cell. Infect. Microbiol..

[44] Hole C.R., Leopold Wager C.M., Mendiola A.S., Wozniak K.L., Campuzano A., Lin X., Wormley F.L. (2016). Antifungal activity of plasmacytoid dendritic cells against *Cryptococcus neoformans* in vitro requires expression of dectin-3 (CLEC4D) and reactive oxygen species. Infect. Immun..

[45] Wozniak K.L., Levitz S.M. (2011). T-Cell and DC Responses to Cryptococcus.

[46] Koguchi Y., Kawakami K. (2002). Cryptococcal infection and Th1-Th2 cytokine balance. Int. Rev. Immunol..

[47] Wozniak K.L., Olszewski M.A., Wormley F.L. (2015). Molecules at the interface of *Cryptococcus* and the host that determine disease susceptibility. Fungal Genet. Biol..

[48] Wormley F.L., Perfect J.R., Steele C., Cox G.M. (2007). Protection against cryptococcosis by using a murine gamma interferon-producing *Cryptococcus neoformans* strain. Infect. Immun..

[49] Specht C.A., Lee C.K., Huang H., Tipper D.J., Shen Z.T., Lodge J.K., Leszyk J., Ostroff G.R., Levitz S.M. (2015). Protection against experimental cryptococcosis following vaccination with glucan particles containing *Cryptococcus* alkaline extracts. mBio.

[50] Specht C.A., Lee C.K., Huang H., Hester M.M., Liu J., Luckie B.A., Torres Santana M.A., Mirza Z., Khoshkenar P., Abraham A. (2017). Vaccination with recombinant *Cryptococcus* proteins in glucan particles protects mice against cryptococcosis in a manner dependent upon mouse strain and cryptococcal species. mBio.

[51] Huang H., Ostroff G.R., Lee C.K., Agarwal S., Ram S., Rice P.A., Specht C.A., Levitz S.M. (2012). Relative contributions of dectin-1 and complement to immune responses to particulate β-glucans. J. Immunol..

[52] Huang H., Ostroff G.R., Lee C.K., Specht C.A., Levitz S.M. (2010). Robust stimulation of humoral and cellular immune responses following vaccination with antigen-loaded β-glucan particles. mBio.

[53] Huang H., Ostroff G.R., Lee C.K., Specht C.A., Levitz S.M. (2013). Characterization and optimization of the glucan particle-based vaccine platform. Clin. Vaccine Immunol..

[54] Ueno K., Kinjo Y., Okubo Y., Aki K., Urai M., Kaneko Y., Shimizu K., Wang D.-N., Okawara A., Nara T. (2015). Dendritic cell-based immunization ameliorates pulmonary infection with highly virulent *Cryptococcus gattii*. Infect. Immun..

[55] Ueno K., Urai M., Takatsuka S., Abe M., Miyazaki Y., Kinjo Y. (2017). Immunization with antigen-pulsed dendritic cells against highly virulent *Cryptococcus gattii* infection: Analysis of cytokine-producing T cells. Methods Mol. Biol..

